# Formulation and Evaluation of Polymeric Spherical Agglomerates-Based Porous Orodispersible Tablets of Cilnidipine

**DOI:** 10.3390/pharmaceutics17020170

**Published:** 2025-01-28

**Authors:** Yahya Alhamhoom, Sanjana S. Prakash, Avichal Kumar, Shivakumar Hagalavadi Nanjappa, Mohamed Rahamathulla, Megha S. Kamath, Syeda Ayesha Farhana, Mohammed Muqtader Ahmed, Thippeswamy Boreddy-Shivanandappa

**Affiliations:** 1 Department of Pharmaceutics, College of Pharmacy, King Khalid University, Al Faraa, Abha 62223, Saudi Arabia; ysalhamhoom@kku.edu.sa; 2 Department of Pharmaceutics, KLE College of Pharmacy, Rajajinagar, Bengaluru 560010, India; sanjanasp307@gmail.com (S.S.P.); avichalk0994@gmail.com (A.K.); meghakamath215@gmail.com (M.S.K.); 3 Department of Pharmaceutics, College of Pharmacy, Qassim University, Buraidah 51452, Saudi Arabia; a.farhana@qu.edu.sa; 4 Department of Pharmaceutics, College of Pharmacy, Prince Sattam Bin Abdul Aziz University, Al Kharj 11942, Saudi Arabia; muqtadernano@gmail.com; 5 Department of Biomedical Science, College of Pharmacy, Shaqra University, Al-Dawadmi Campus, Al-Dawadmi 11961, Saudi Arabia; drswamy@su.edu.sa

**Keywords:** cilnidipine, polymeric spherical agglomerates, bioavailability enhancement, orodispersible tablets, in vitro study

## Abstract

**Background/Objectives:** Cilnidipine (CIL) is a calcium channel blocker that exhibits low bioavailability (~13%) due to poor aqueous solubility and extensive pre-systemic gut wall metabolism. The current study aimed to enhance the oral bioavailability of CIL by formulation of polymeric spherical agglomerates (CILSAs)-based orodispersible tablets (ODTs). **Methods:** Eight different batches of CILSAs were prepared by a crystallo-co-agglomeration technique using different proportions of hydrophilic polymers like hydroxy propyl methyl cellulose E50, polyvinyl pyrrolidone K30, or polyethylene glycol (PEG) 6000 as carriers. Fourier transform infrared spectroscopy (FTIR) of CILSAs proved the chemical integrity of CIL in SAs, while scanning electron microscopy revealed the spherical shape of CILSAs. **Results:** Differential scanning calorimetry and powder X-ray diffraction studies confirmed that CIL was rendered more amorphous in CILSAs. CILSAs displayed good flow behavior, high percentage yield, and high drug loads. The batch F4 composed of PEG 6000 emerged as the optimized batch as it displayed high percentage dissolution efficiency (57.01 ± 0.01%), which was significantly greater (*p* < 0.001) compared to CIL (26.27 ± 0.06%). The optimized formulation of CILSAs was directly compressed into ODTs that were rendered porous by vacuum drying. The optimized formulation of porous ODTs (T3) displayed low friability (0.28 ± 0.03%), short disintegration time (6.26 ± 0.29 s), and quicker dissolution (94.16 ± 1.41% in 60 min) as compared to marketed tablet Cildipin^®^ 10 mg (85 ± 2.3%). **Conclusions:** Thus, porous ODTs of CILSAs can rapidly release the drug, bypass gut metabolism, enhance oral bioavailability, and improve CIL’s therapeutic effectiveness for angina and hypertension.

## 1. Introduction

Angina and hypertension are interrelated cardiovascular conditions that significantly impact global health. Angina, characterized by chest pain or discomfort, arises from reduced blood flow to the heart muscles, often due to coronary artery disease. Hypertension, characterized by persistently high blood pressure, increases the risk of angina, heart attack, stroke, and chronic kidney disease. Hypertension is the most important risk factor for cardiovascular morbidity and mortality. An estimated 57% of strokes and 24% of coronary artery disease-related deaths are caused by hypertension. The prevalence of hypertension in adults ranges from 20 to 40% in cities and 12 to 17% in rural areas. The prevalence of hypertension is expected to rise from 118 million in 2000 to 214 million by 2025, with approximately equal numbers of men and women affected [1,2].

Cilnidipine (CIL) is a newer dihydropyridine calcium antagonist used to manage cardiovascular conditions such as angina and hypertension. It acts by inhibiting calcium ion entry into the heart and blood vessel walls, leading to relaxation and dilation of smooth muscles, thereby “improving blood flow and reducing strain on the heart” [3]. The drug is unique compared to other L-type calcium channel blockers or even other antihypertensives, as it could be selected according to the pathophysiological condition of a patient [4]. The drug is characterized by a short half-life (20.4 min), high molecular weight (492.52 g/mol), and a high protein binding of 98% [5]. CIL is categorized as Biopharmaceutical Classification System (BCS) Class II due to its low aqueous solubility (≤2 mg/mL), which leads to poor dissolution. In addition, the drug exhibits poor flow properties that are likely to pose processing or manufacturing issues. The dissolution of CIL is said to be the rate-limiting step in drug absorption following oral administration [6]. Considering this, attempts have been made in the past to resolve the solubility issues of CIL by formulation of β inclusion complex [7], solid dispersion [8], nanoparticles [9], and nanosuspension [10]. In addition to the poor solubility, CIL undergoes extensive pre-systemic metabolism by CYP3A4 enzymes within the gut wall, resulting in low oral bioavailability (13%).

Considering these limitations of CIL, the current study endeavors to develop polymeric spherical agglomerates of CIL (CILSAs) using various hydrophilic polymers, including hydroxyl propyl methyl cellulose (HPMC) E50, polyvinyl pyrrolidone (PVP) K 30, and polyethylene glycol (PEG) 6000. The study further plans to compress the CILSAs into orodispersible tablets (ODTs) that would be vacuum-dried to render the tablets porous. Crystallo-co-agglomeration is a promising technique that transforms complex, needle-shaped crystals of poorly soluble drugs with compressibility problems into free-flowing polymeric SAs, which has the potential to significantly ease downstream processing [11]. To resolve the poor flow properties and solubility issues, the technique is used to convert crystalline material to SAs that possess better solubility and micrometric properties in terms of flowability, packability, and compressibility using hydrophilic polymers as carriers [12,13,14]. In this context, the present study primarily aims to produce SAs to improve the dissolution and flow properties of CIL using different hydrophilic polymers such as hydroxy propyl methyl cellulose E50, polyvinyl pyrrolidone K 30 or polyethylene glycol (PEG) 6000. The optimized SAs of CIL are compressible into orodispersible tablets (ODTs), which would quickly disintegrate into the constituent SAs in the oral cavity and generate a pulsed dose of CIL. Thus, the generated pulsed dose is likely to saturate the metabolizing enzymes in the gut wall, evading the pre-systemic elimination of CIL thereby enhancing drug absorption and bioavailability [15]. The proposed approach of SAs-based ODTs for CIL introduces a novel strategy to address gut wall metabolism and enhance bioavailability. This first-of-its-kind innovative approach focuses on formulating ODTs that rapidly disintegrate to deliver a pulsed dose of CIL that would increase drug absorption. The present study was taken up because no such studies have explored this novel approach to improve the absorption of CIL by formulation of polymeric SAs to boost the therapeutic efficacy.

## 2. Materials and Methods

### 2.1. Materials

Cilnidipine was purchased from Yarrow Chem Pvt. Ltd., Mumbai, India. Glyceryl monostearate (GMS), polyvinyl pyrrolidine K-30 (PVP K 30), polyethylene glycol 6000 (PEG 6000), and hydroxy propyl methyl cellulose E 50(HPMC) were purchased from Central Drug House Pvt. Ltd. (New Delhi, India) and Hi-Media Lab Pvt Ltd. (Mumbai, India). Croscarmellose sodium was from Roquette (I) Pvt. Ltd., Avicel^®^ PH 102 was from FMC (I) Pvt. Ltd., while cross-povidone K30 and Soluplus^®^ was from BASF. S.D. Fine Chemicals, Mumbai, India, supplied the rest of the analytical grade chemicals and reagents.

### 2.2. Solvent System Selection for Spherical Agglomerates Preparation

The selection of the solvent system was conducted by screening a number of solvents required for the process of agglomeration. We determined the solubility of CIL in various solvents such as ethanol, dichloromethane (DCM), and water using the shaker flask method. An excess amount of CIL was added to 3 mL of each solvent until saturation, and the resulting dispersion was allowed to stand for 48 h at room temperature with intermittent shaking. The saturated solution obtained was centrifuged at 3000 rpm for 10 min at 25 °C, and the supernatant was collected. The supernatant liquid was filtered using Whatman filter paper (Maidstone, UK), appropriately diluted, and the content of CIL was determined spectrophotometrically at 241 nm (Shimadzu 1900i UV Visible spectrophotometer, Kyoto, Japan) using the calibration curve constructed in the same solvent system [16].

### 2.3. Preparation of Spherical Agglomerates

**Figure 1:**
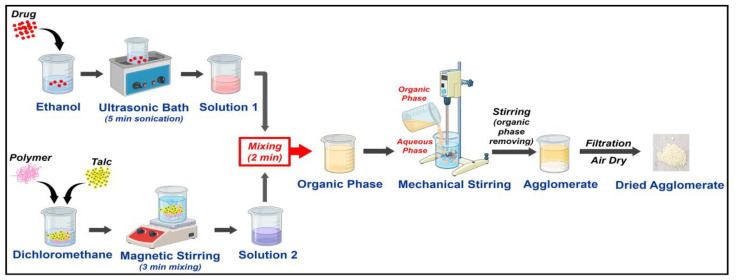
Flow chart illustrating preparation of spherical agglomerates.

Crystallo-co-agglomeration was employed to produce SAs of CIL using different polymers as carriers. CIL was dissolved in ethanol with sonication (GT Sonic Professional Ultrasonic Bath, Shenzhen, China) for 5 min to ensure complete dissolution. In the other beaker, different amounts of polymers, as listed in Table 1, were dissolved in DCM under magnetic stirring (2 mL) (Magnetic Stirrer, Remi Motors, Mumbai, India). To the polymeric solution, a fixed amount of talc was added, and stirring continued for another 3 min to obtain a homogenous dispersion. To the resulting dispersion, the alcoholic solution of the drug was added, and stirring continued for 2 min. The resulting organic polymeric dispersion containing CIL was slowly added to the aqueous phase comprising 120 mL of water with continuous stirring under a mechanical stirrer (RQ-127/A, Remi motors) at 1200 rpm. The mixing was continued for a period of 1 h to enable complete evaporation of the organic solvents. The spherical agglomerates (SAs) produced were filtered and left to air dry overnight at room temperature [17,18]. The SAs were finally stored in airtight aluminum pouches kept in a desiccator. A detailed illustration of spherical agglomerates is shown in Figure 1.

### 2.4. Characterization of Spherical Agglomerates

#### 2.4.1. FTIR Spectroscopy (FTIR)

FTIR spectroscopy was carried out to check for possible interaction between CIL and other excipients used during the process. FTIR spectra of CIL, physical mixture, and the SAs were acquired in a Jasco FTIR (460Plus) spectrometer. The samples were intimately mixed with potassium bromide in a glass mortar and pestle. The mixtures obtained were then loaded in an IR diffuse reflectance sampler and exposed to infrared radiation. The samples were scanned between 500 and 3500 cm^−1^ after running a blank with potassium bromide to detect the drug’s distinctive peak [19]. The characteristic peaks of the SAs were compared with those of CIL that acted as a reference.

#### 2.4.2. Percentage Yield

The yield of the developed SAs was calculated in order to determine the amount of product recovered following the whole production process. The weight of SAs of individual batches was recorded, and the percentage practical yield of each batch was determined using Equation (1) [20]. The percent practical yield for each individual batch was determined in triplicate, and the results have been provided as mean ± SD (standard deviation).

(1) $$\%Practical\;Yield=\frac{Weight\;of\;obtained\;agglomerates}{Initial\;Weight\;of\;drug\;and\;polymer\;used}\times 100$$

#### 2.4.3. Drug Content

The drug content of prepared SAs was determined by carefully weighing the product equivalent to 10 mg and dispersing it in ethanol in a volumetric flask, followed by sonication for 15 min to ensure complete dissolution of the SAs. The dispersion obtained was filtered using Whatman filter paper (Maidstone, UK). The filtrate obtained was appropriately diluted with phosphate buffer pH 6.8 containing tween 80 (0.5% *v*/*v*) in a volumetric flask. The absorbance of the resulting solution was determined at 241 nm to determine the drug content using the slope of the standard calibration curve constructed in the same solvent system. The assay was determined in triplicate, and the results were expressed as mean ± SD [20].

#### 2.4.4. Pre-Compression Parameters (Micromeritic Properties)

The angle of repose (AOR), bulk density (BD), tapped density (TD), Carr’s index, and Hausner ratio were used to characterize the flow characteristics of CIL and SAs produced. Tapped density was assessed by loading 20 g of the sample into a 50 mL measuring cylinder. The cylinder was tapped at 50 strokes per minute to obtain a consistent volume in a tap density apparatus (Campbell Electronics, Mumbai, India). Bulk density was determined by the three-tap method by taking the same weight of sample in the measuring cylinder and tapping three times in the same equipment. The TD and BD were determined from the final volumes using Equations (2) and (3), respectively. From the results of the tapped density and bulk density, the compressibility index (Carr’s index) and Hausner ratio of SAs were computed using Equations (4) and (5), respectively [21,22].

(2) $$Tapped\;density=\frac{Mass\;(g)}{Tapped\;volume\;(mL)}$$

(3) $$Bulk\;density=\frac{Mass\;(g)}{Bulk\;volume\;(mL)}$$

(4) $$Compressibility\;index\%=\frac{Tapped\;density\left(g\right)-Bulk\;density\;(g)}{Tapped\;density}\times 100$$

(5) $${Hausner}^{’}sratio=\frac{Tapped\;density}{Bulk\;density}$$

The fixed funnel method was utilized to obtain the angle of repose, in which a funnel was affixed at a constant height of 2.5 cm above a horizontal surface [23]. The samples were allowed to flow gently through the fixed funnel until the tip of the cone-shaped assemblage just touched the lower funnel tip. Monitoring the height and diameter of the funnel allowed to determine the angle of repose using Equation (6).

(6) $$\theta =\tan^{-1}\frac{height}{radius}$$

#### 2.4.5. Scanning Electron Microscopy

A scanning electron microscope (SEM) was used to examine the surface topography and morphological features of the SAs. The samples were initially subjected to a vacuum for 15 min to remove the surface artifacts. Then, the samples were mounted on a double-sided adhesive tape and coated with gold to render them electrically conductive. Subsequently, the coated gold samples were exposed to the field of emission and examined under a scanning electron microscope (Tescan-Vega3 LMU). After that, the samples were randomly scanned and photomicrographs were obtained under 15–18 kV acceleration voltage. To study the surface morphology, the SAs were observed at appropriate magnification before capturing images of the samples [24,25].

#### 2.4.6. Particle Size

The Malvern Mastersizer 2000 particle size analyzer was employed to analyze the particle size distribution through laser diffraction. The SAs were uniformly dispersed in water by subjecting them to sonication at 600 W for 5 min prior to measurement to uniformly disperse the SAs. The Mastersizer 2000 was used to ascertain particle size distribution conforming to the 50th (D 50%) and 90th (D 90%) percentile of particles and span values. The presented results show three determinations’ mean and standard deviation (SD) [26]. A plot of volume density versus average particle size would be directly obtained from the measurement. The volume/surface mean diameters were computed as per Equation (7) [27].

(7) $$Volume\;surface\;Mean\;Diameter\left(d_{vs}\right)=\frac{\sum nd^{3}}{\sum nd^{2}}$$

#### 2.4.7. Differential Scanning Calorimetry (DSC)

DSC is a thermoanalytical technique that is utilized to characterize the solid state of the drug in the samples. The study helps to identify possible solid-state transformations during the formulation process. DSC measurements were performed in a differential scanning calorimeter (Netzsch STA 449 F5 Jupiter, Selb, Germany). The carefully weighed sample was sealed in an aluminum pan and heated under nitrogen flow (20 mL/min) at a heating rate of 30 °C/min in the range of 10 to 150 °C [28]. The degree of crystallinity (Xc) of physical mixtures and formulations with respect to CIL was calculated using Equation (8).

(8) $$Xc=\frac{∆Hm}{(1-w)\cdot ∆H{}^{\circ}m}$$

The measured heat of fusion is ∆Hm; the heat of fusion of 100% crystalline CIL is ∆H°m; the weight fraction of the sample in the carrier matrix is w.

#### 2.4.8. X-Ray Powder Diffraction

In addition to DSC, XRD was used to examine the solid state of CIL in the samples. XRD patterns of the samples were analyzed by a Bruker D8 Advance X-ray diffractometer (Billerica, MA, USA) with a 2.2 kW X-ray source of Cu anode with fine focus ceramic X-ray tube operated at 40 kV voltages and 40 mA current at 1.6 kW power [29]. The relative degree of crystallinity (RDC) with respect to CIL was computed using Equation (9).

(9) $$RDC=\frac{I_{sample}}{I_{refer}}\times 100$$

The term “*I_sample_*” refers to the peak height of the sample with the highest intensity, while “*I_refer_*” refers to the peak height of CIL at the same 2θ.

#### 2.4.9. In Vitro Release Study of Spherical Agglomerates

The dissolution studies were carried out in 900 mL phosphate buffer pH 6.8 containing tween 80 (0.5% *v*/*v*) in a USP dissolution apparatus type-II (Electrolab TDT-08L, Mumbai, India). The studies were performed at a bath temperature of 37 ± 0.5 °C and 50 rpm stirring speed. A total of 5 mL of samples were withdrawn at regular time intervals of 0, 10, 20, 30, 40, 50, and 60 min. Subsequently, the bath was replaced with 5 mL of fresh buffer equilibrated to the same temperature. The samples withdrawn were filtered and assayed after suitable dilutions in a UV spectrophotometer at a wavelength of 241 nm to determine the drug release amount at different time intervals [30]. A plot of the percentage of the drug dissolved at different time points was plotted against time to obtain the dissolution profiles. The percentage dissolution efficiency (%DE) for each batch of SAs and CIL was calculated based on the area under the drug release profile curve as per Equation (10).

(10) $${\%DE}_{=}\frac{\int_{t_{0}}^{T}y_{t}(dt)}{y_{100}\times T}\times 100$$

### 2.5. Formulation of Rapid Orodispersible Tablets

The optimized batch of CILSAs was directly compressed to ODTs using directly compressible excipients. The tablet formulation contains the SAs, super disintegrants, and other directly compressible materials, including a sweetening agent and pore-forming agent (Table 2). The agglomerates and excipients were thoroughly mixed in a poly bag before passing through a no. 44 sieve prior to compression into tablets. The compression was performed using a 6 mm flat punch in a rotary tablet press (Model RSB-4, Rimek mini press) to obtain tablets weighing 70 to 80 mg to a hardness of 3–4 kg [24]. The compressed tablets containing ammonium bicarbonate were subjected to vacuum drying in a vacuum oven (Servewell Instruments Pvt. Ltd., Bengaluru) at 60 °C and 250 mm Hg overnight to ensure complete removal of ammonium bicarbonate and formation of porous tablets [31].

### 2.6. Evaluation of Orodispersible Tablets

#### 2.6.1. Bright-Field Microscopy

Bright-field microscopy, which employs visible light to illuminate samples, was utilized to assess the surface characteristics and topographical features of both porous ODTs and ODTs. The prepared ODTs were placed onto a glass slide and observed using a microscope equipped with a bright-field objective and condenser (Labomed Vision 2000). Images of the samples were captured at 100× magnifications [25]. 

#### 2.6.2. Post-Compression Parameters

The ODTs were examined for various tablet characteristics such as weight variation, hardness, thickness, friability, wetting time, disintegration, content uniformity, and in vitro dissolution in order to determine if they passed the compendial norms.

##### Weight Variation

A random sample of 20 ODTs from each batch was selected, and the weight of each tablet was recorded individually to check for weight variations using an electronic balance (Analytical Balance, BL-220H Shimadzu, Kyoto, Japan). The weight of each tablet was subsequently determined and compared to the average value. The result outcomes were expressed as mean ± SD [32].

##### Hardness

Hardness is a measure of the resistance of ODTs to mechanical shocks during processing and transportation. A hardness tester (Monsanto, Missouri, USA) was used to determine the hardness, which was expressed as kg/cm^2^. Ten tablets were randomly selected from each batch to examine the hardness of the tablets. The result outcomes were expressed as the mean ± SD of 10 determinations [32]. 

##### Thickness

The thickness of the ODTs was determined using a digital Vernier caliper (Mitutoyo Corporation, Tokyo, Japan). Uniformity in tablet thickness is a key parameter that determines the ease of blister packing. The results of the tablet thickness were expressed as the mean ± SD [32].

##### Friability

Friability was determined as a reduction in tablet weight after being subject to wear and tear. The tablets equivalent to 6.5 g were taken for evaluation in accordance with IP in a friabilator (Electrolab EF-20). Firstly, the initial weight of tablets before loading into the friabilator was recorded (Wₒ). The friabilator containing tablets was rotated at a speed of 25 rpm for 4 min, where the tablets were dropped from a height of 6 inches. Later, the tablets were reweighed (W) after 100 revolutions, and differences in weight between the tablets before and after the test were noted down. The reduction in the weight of tablets as a result of breaking or attrition was recorded and is referred to as the percentage friability. The outcomes were given as the mean ± SD [32,33]. The percentage friability (% F) was calculated using Equation (11).

(11) $$\%F=\frac{W_{o}-W}{W_{o}}\times 100$$

##### Wetting Time

A piece of tissue paper was rolled twice and placed in a 5.5 cm diameter Petri plate. About 10 mL of distilled water containing a dye maintained at a temperature of 37 °C was added to the plate. The time taken for the tablet placed on the tissue paper to get wet was recorded as the total wetting time. The experimental outcomes were expressed as the mean ± SD [34].

##### Disintegration Test

Disintegration is the process of breaking down a tablet into minimal fragments. The disintegration time of a tablet was determined using a disintegration test apparatus (Electrolab ED-2L, Mumbai, India). Six tablets were dropped into each of the six tubes of the disintegration apparatus, where distilled water was used as a disintegrating medium that was maintained at a temperature of 37 ± 0.5 °C. A disk was placed over the tablet in each of the tubes during the test. Time taken for the complete disintegration of the tablet was noted down, and the results were expressed as the mean ± SD [35].

##### Drug Content

A content uniformity test was performed on 10 tablets selected randomly to ensure uniform potency. The drug content was determined by triturating the tablets to ascertain the assay of CIL in the formulation. The powder containing 10 mg of CIL was dissolved in 10 mL of ethanol. The resulting dispersion was sonicated and filtered using Whatman filter paper (Maidstone, UK). The filtrate obtained was appropriately diluted with phosphate buffer pH 6.8 containing tween 80 (0.5%). The absorbance was determined at 241 nm in a visible UV spectrophotometer to determine the drug content using the slope of the standard calibration curve constructed in the same solvent system. The results of drug content were determined and expressed as mean ± SD [36].

#### 2.6.3. In Vitro Dissolution Studies of Prepared Orodispersible Tablets

In vitro dissolution studies were carried out in a USP dissolution test type II apparatus (Electrolab TDT-08L, Mumbai, India). The dissolution test was conducted in 900 mL phosphate buffer of pH 6.8 containing tween 80 (0.5%), which was stirred at 50 rpm and maintained at a temperature of 37 ± 0.5 °C. About 5 mL of samples were withdrawn every 10 min for a period of 1 h and replaced with fresh buffer. The quantity of drug dissolved at various time points was determined by measuring the absorbance at 241 nm. To determine the dissolution profile, the amount of drug release was plotted versus time [37]. The similarity factor (f_2_) and dissimilarity factor (f_1_) were determined to compare the dissolution profiles of ODTs with the constituent SAs using Equations (12) and (13), respectively.

(12) $$f_{1=}\frac{\left\{\left\{\sum_{t=1}^{n}\left|Rt-Tt\right|\right\}\right\}}{\sum_{t=1}^{n}Rt}\times 100$$

(13) $$f_{2}=50\times \log\left[{\left\{1+\frac{1}{n}\sum_{r=1}^{n}Wt\left(Rt-Tt\right)\right\}}^{-0.5}\times 100\right]$$

where *Tt* is the average percentage of drug dissolved in the test formulation; *n* is the number of observations; *Rt* is the average percentage of drug dissolved in the reference formulation. The weight factor, abbreviated *Wt*, is typically considered to be 1.

The drug release profile of the ODT developed was compared with that of the marketed tablet (Cildipin^®^ 10 mg) [8,38].

### 2.7. Stability Study

The selected ODTs were subjected to stability studies over three months under controlled conditions of 25 ± 2 °C with 60 ± 5% relative humidity (RH) and 40 ± 2 °C with 75 ± 5% RH for accelerated conditions [39]. At the end of the study, critical parameters such as weight variation, friability, hardness, disintegration time, drug content, and drug release profile were evaluated. The results were then compared with the initial data recorded before the stability testing.

### 2.8. Statistical Analysis

The results generated during the studies were analyzed in Graphpad Prism version 9. Statistical analysis was performed by one-way analysis of variance and *t*-test in Graphpad Instat 5 software (GraphPad Software, Inc., La Jolla, CA, USA). A *p*-value of less than 0.05 was considered to be statistically significant. The error bars represent the standard deviation (SD).

## 3. Results

### 3.1. Screening of Solvents for Spherical Agglomerates Preparation

Solubility studies of CIL in different solvents indicated that the drug solubility was 20.00 ± 0.03 mg/mL, 16.2 ± 0.02 mg/mL, and 0.001 ± 0.02 mg/mL in ethanol, DCM, and water, respectively.

### 3.2. Evaluation of Prepared Sphere Agglomerates

#### 3.2.1. FTIR Spectroscopy

**Figure 2:**
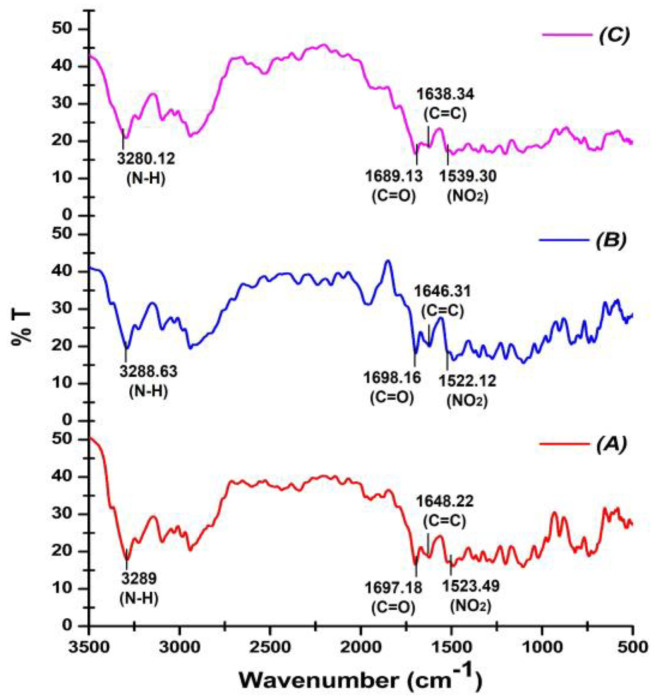
FTIR spectra of CIL (A), PM (B), and spherical agglomerates (C).

CIL displayed characteristic peaks at 3289.0 cm^−1^, 1697.18 cm^−1^, 1648.22 cm^−1^, and 1523.49 cm^−1^. The physical mixture (PM) exhibited all the characteristic peaks of CIL, ruling out the possibility of any chemical interaction between the drug and excipients. Similarly, the drug characteristic peaks were clearly evident in SAs, indicating the chemical integrity of CIL during the production of SAs, as shown in Figure 2 and Table 3. 

#### 3.2.2. Percentage Yield

The percentage yield was calculated for formulations F1-F8, which were found to range from 66.82 ± 0.08% to 84.02 ± 0.04%, as shown in Table 4. The percentage yield was highly dependent on the polymers employed to produce the SAs.

#### 3.2.3. Drug Content

The drug content was found to depend on the type of polymeric carrier used to prepare the SAs. The drug content was found in the range of 83.65 ± 0.42 to 97.49 ± 0.29%, with the highest percentage drug content of 97.49 ± 0.29% in F4, as displayed in Table 4.

#### 3.2.4. Micromeritic Property

The angle of repose, Carr’s index, and Hausner’s ratio for SAs were found to range from 22.4 ± 0.03° to 24.2 ± 0.07°, 11.66 ± 0.01 to 11.66 ± 0.01, and 1.11 ± 0.03 to 1.18 ± 0.05, respectively. The prepared polymeric SAs of CIL (F1-F8) have acceptable micromeritic properties, as shown in Table 5.

#### 3.2.5. Scanning Electron Microscopy

**Figure 3:**
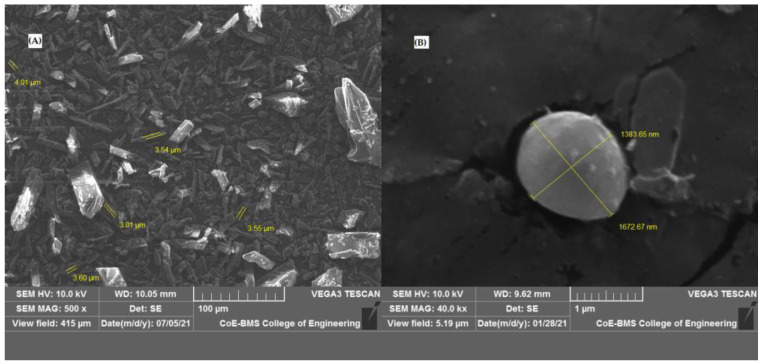
SEM images of Cilnidipine (**A**) and Spherical Agglomerate of optimized batch F4 (**B**).

SEM micrographs revealed that the bulk CIL displayed long, needle-shaped crystals that were clearly visible under a magnification of 500×. On the contrary, the SAs (F4) were found to be spherical with smooth surfaces in SEM photographs only under a magnification of 40,000×, as displayed in Figure 3.

#### 3.2.6. Particle Size

**Figure 4:**
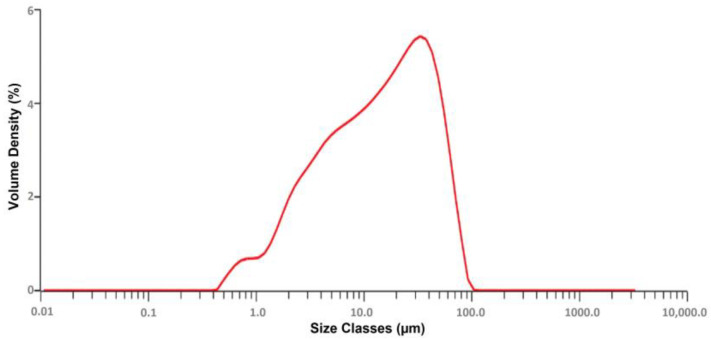
Particle size distribution curve of the optimized batch of Spherical Agglomerate F4.

The particle size distribution of F4 is captured in Figure 4. Particle size was found to range from 0.523 to 98.11 µm, the volume median diameter (D10%) of the agglomerates was found to be 2.31 µm, and (D90%) of the agglomerates were around 48.85 µm. The volume surface diameter for F4 was found to be 5.78 µm.

#### 3.2.7. Differential Scanning Calorimetry

**Figure 5:**
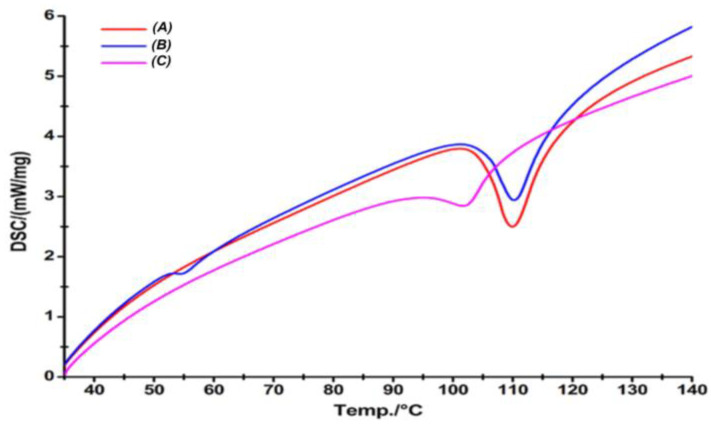
Differential Scanning Calorimetry of CIL (A), PM (B), and Spherical Agglomerates (C).

The DSC scan of the drug demonstrated a strong endothermic peak at 110 °C with an enthalpy of fusion (∆Hf) value of 90.16 J/g. The physical mixture, too, displayed an endothermic peak at 109 °C with reduced intensity (∆Hf value of 21.25 J/g), indicating the semicrystalline nature of the CIL in the mixture, which indicated that nearly 47.15% of CIL was in crystalline form. However, the drug peak was substantially reduced in intensity in CILSA (∆Hf of 7.6 J/g), indicating a reduction in crystallinity of CIL to 16.8%, as shown in Figure 5.

#### 3.2.8. X-Ray Powder Diffraction

**Figure 6:**
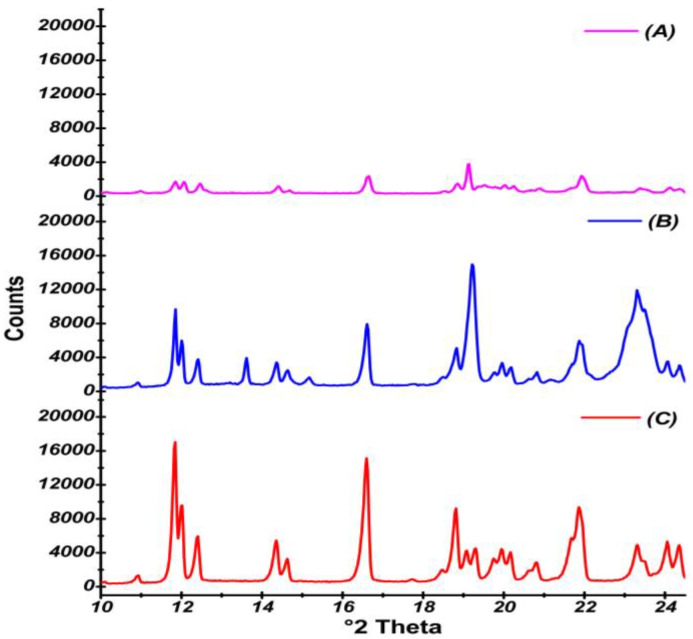
X-ray powder diffraction of Spherical Agglomerates (A), PM (B), CIL (C).

The PXRD patterns obtained for the samples analyzed are illustrated in Figure 6. The PXRD pattern clearly showed six distinct, well-defined high-intensity distinct peaks at 2θ values at 11.84°, 12.41°, 14.35°, 16.59°, 18.83°, and 21.86° indicating the crystalline nature of the CIL. Likewise, the diffractogram of the physical mixture exhibited low-intensity peaks at 11.82°, 12.41°, 14.38°, 16.58°, 18.83°, and 21.84° indicating a decrease in the drug crystallinity. In contrast, the diffractogram of F4 displayed a total of 6 characteristic low-intensity peaks of CIL at 11.82°, 12.41°, 14.38°, 16.58°, 18.83°, and 21.84° that suggested further drop in the drug crystallinity.

#### 3.2.9. In Vitro Release Study of Spherical Agglomerates

**Figure 7:**
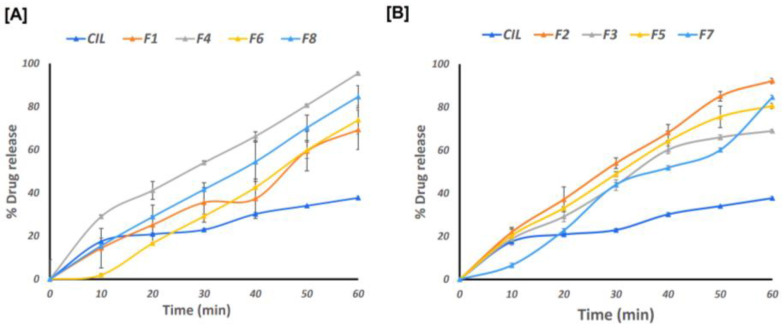
Drug release (%) vs. time (min) for formulation CIL; F1; F4; F6; F8 in (**A**) and CIL; F2; F3; F5; F7 in (**B**).

The percentage drug release and dissolution efficiency (%DE) of SAs is shown in Figure 7 and Table 6. CIL was found to exhibit a release of 37.73 ± 0.89% at the end of 60 min, which corresponded to a %DE of 26.27 ± 0.06. The in vitro release profile of the different formulations of SAs was found to range from 56.37 ± 0.018 to 95.50 ± 0.012%, as depicted in Figure 7A,B. Likewise, the %DE for different batches of SAs was found to range from 37.57 ± 0.08 to 57.01 ± 0.01.

### 3.3. Formulation of Orodispersible Tablets

The SAs of batch F4 were mixed directly with compressible materials such as MCC, mannitol, croscarmellose sodium, crospovidone, ammonium bicarbonate, magnesium stearate, and talc, listed in Table 2, and compressed into ODTs.

### 3.4. Evaluation of Orodispersible Tablets

#### 3.4.1. Bright Field Microscopy

**Figure 8:**
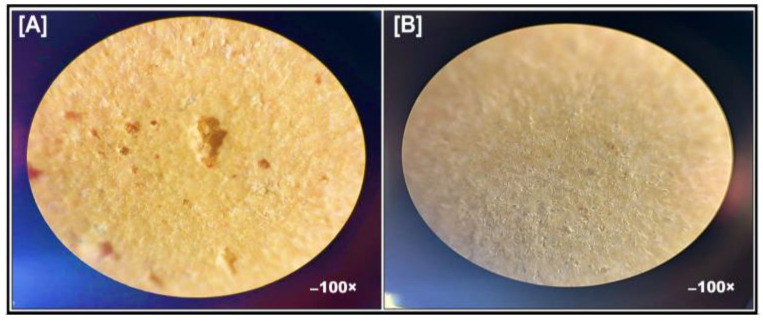
Microscopic images of (**A**) Porous ODT [T3], (**B**) ODT [T4] at 100× magnification.

All the tablets produced by direct compression displayed a smooth surface without any visible deformities. However, surface morphology and topography analysis under a bright-field microscope at 100× magnification revealed that the surface topography of ODTs in batches T1 and T3 displayed hollow and well-defined pores (Figure 8A). In contrast, ODTs prepared in batches T2 and T4 did not display any visible pores (Figure 8B).

#### 3.4.2. Post-Compression Parameters

**Figure 9:**
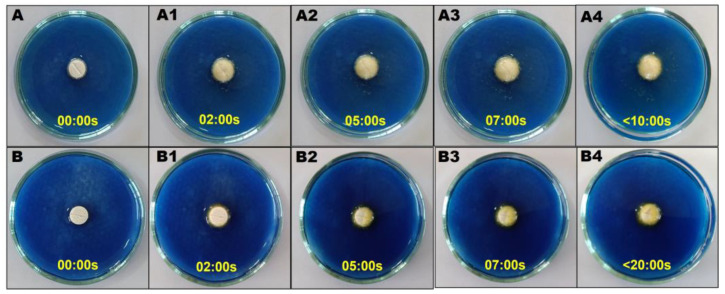
Images illustrating the wetting time of Porous ODTs [T3] of SAs (**A**) and ODTs [T4] of SAs (**B**).

The porous ODTs and ODTs of CIL and SAs manufactured by direct compression were found to comply with the pharmacopeia limits in Table 7. The wetting time for both porous and nonporous ODTs was found to be less than 30 s. The captured pictures of the wetting time at different time points of ODTs are highlighted in Figure 9 and Supplementary Video recording files (Videos S1 and S2). The wetting time of 19.82 ± 0.04 s and 6.22 ± 0.03 s was observed for batches T1 and T3, respectively. On the other hand, the wetting time of 21.68 ± 0.08 s and 7.61 ± 0.06 s was observed for batches T2 and T4. Likewise, batches T1 and T3 displayed a DT of 21.12 ± 0.01 s and 6.26 ± 0.29 s, respectively, while T2 and T4 displayed a DT of 25.12 ± 0.06 s and 8.26 ± 0.21 s, respectively.

#### 3.4.3. In Vitro Release Study of Prepared Orodispersible Tablets

**Figure 10:**
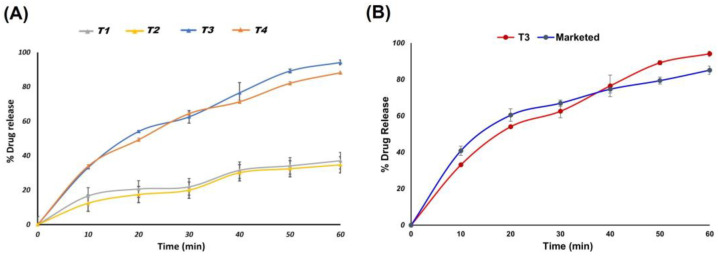
(**A**) Comparative dissolution profiles of porous ODTs and ODTs (**A**) and porous ODTs (T3) vs. marketed tablet (**B**).

The CILSAs that contained 10 mg of the CIL that displayed the optimum release pattern were chosen to produce ODT. The drug release rate was found to be considerably higher for tablets of CILSAs that were rendered porous, which indicated that the release of the drug from tablets was influenced by the porosity of the tablet. Likewise, the porous tablets exhibited a better drug release compared to their nonporous counterparts, confirming the effect of a subliming agent induced in the formulation. The porous tablets of CILSAs displayed the highest drug release of 94.16 ± 1.41%, which was significantly higher (*p* < 0.002) than the nonporous ODTs of SAs (84.75 ± 1.69%). Likewise, the drug release from porous ODTs of CIL (37.10 ± 0.37%) was found to be significantly higher (*p* < 0.001) than the nonporous ODTs of CIL, displaying a drug release of 34.70 ± 0.27%, as portrayed in Figure 10A. The best drug release profile of porous ODTs (T3) displayed superior drug release compared to the marketed tablet (85 ± 2.3%), as shown in Figure 10B.

### 3.5. Stability Study

The ODTs (T3) were analyzed for various physicochemical and post-compression parameters after completing the stability studies. The findings from these studies are summarized in Table 8.

## 4. Discussion

Crystallo-co-agglomeration techniques transform poorly soluble drugs into spherical, free-flowing particles where the drug would be embedded in an amorphous state in a hydrophilic carrier, thereby improving the solubility and dissolution rate. The process results in directly compressible agglomerates of several poorly compressible drugs [40]. By incorporating suitable hydrophilic additives during the development of SAs, the rate at which the drug dissolves from the agglomerates or compacts can be improved. This procedure produces SAs that are simple to process and “ready-to-compress”, which reduces the associated compressibility issues. Spherical crystallization is one of the innovative methods that would enhance the bioavailability of medications that display poor aqueous solubility. In addition, agglomeration is one of the promising techniques that have the potential to mask the obnoxious taste of drugs like CIL [41].

The selection of solvent plays a major role in the formulation of SAs, which influences the overall agglomeration process [42]. The choice of this solvent system for crystal agglomeration depends on the miscibility of the solvents and the solubility of the drug in each of the solvents [42]. Based on the preliminary studies, ethanol was identified as a good solvent for CIL. Water was chosen as an anti-solvent, considering the poor aqueous solubility of CIL, while dichloromethane was used as a bridging liquid. The choice of solvent plays a crucial role as it influences the yield and rate of the agglomeration process, as well as the strength of the formed SAs. The SAs formulated using PEG 6000 as a carrier were found to display significantly higher (*p* < 0.0005) drug content compared to other batches of SAs. Similarly, the percentage yield was found to be significantly higher (*p* < 0.0001) for batch F4, which was constituted of PEG 6000. The lower yield in some batches may be attributed to the loss of either the drug or the carrier during the formulation process. Among all formulations, F4 prepared from PEG 6000 displayed significantly higher (*p* < 0.0005) drug content compared to other batches of SAs. The poor drug loading noted with other polymers can be likely due to the failure of the polymers to entrap CIL owing to the poor solubility of CIL in the polymer. Better entrapment is likely when the drug has a good solubility in the polymer.

The SAs of batch F4 were found to exhibit superior flow properties compared to CIL. For instance, the angle of repose of SAs in batch F4 was found to be 23.01 ± 0.03°, which was significantly less (*p* < 0.0001) compared to CIL (31.8 ± 0.02°). Likewise, Carr’s index for the same batch was found to be 11.66 ± 0.01%, which was significantly less (*p* < 0.0001) compared to CIL (33.6 ± 0.08%). Similarly, Hausner’s ratio for F4 of SAs was found to be 1.11 ± 0.03, which was significantly less (*p* < 0.0005) compared to CIL (1.52 ± 0.06). The results obtained conclusively suggested an improvement in compressional and flow characteristics of the SAs constituted of PEG 6000. The findings indicated that SAs (F4) were found to exhibit good flow properties compared to CIL as such.

The FTIR spectrum for CIL displayed prominent peaks at 3289.0 cm^−1^, 1697.18 cm^−1^, 1648.22 cm^−1^, and 1523.49 cm^−1^. The peak obtained at 3289.0 cm^−1^ can be assigned to N-H stretching vibration, while the other peaks observed at 1697.18 cm^−1^ can be attributed to C=O stretching, the peak observed at 1648.22 cm^−1^ can be related to C=C stretching, and the peak obtained at 1523.49 cm^−1^ can be attributed to NO_2_ stretching. Previous investigations endorse FTIR peaks of CIL at 3288.63, 1697.36, 1647.21, and 1523.76 cm^−1^, authenticating the sample of the drug [37,43]. The presence of all of the prominent peaks of CIL in a physical mixture indicated that the drug was chemically intact in the physical mixture. Similarly, the drug characteristic peaks were apparent in the SAs at 3280.12 cm^−1^, 1689.13 cm^−1^, 1638.34 cm^−1^, and 1539.30 cm^−1^, indicating the chemical integrity of CIL in SAs. The results obtained clearly ruled out any possible interaction of CIL with any of the excipients during the process of formulation.

DSC is used as a vital thermoanalytical tool to characterize the solid state of the drug in polymer. The DSC scan of the drug demonstrated a strong endothermic peak at 110 °C with an enthalpy of fusion (∆H_f_) value of 90.16 J/g, which corresponds to its melting transition temperature of 109 °C, confirming from previous literature [10]. The observed ∆Hf value for CIL was found to be similar to the value reported in the literature [6,10]. The physical mixture also displayed an endothermic peak at 109 °C at reduced intensity, indicating the semicrystalline nature of the drug in the mixture but with a reduced ∆H_f_ value of 21.25 J/g, which indicates that nearly 47.15% is in crystalline form. The data infer that nearly 53% of the drug would be soluble in the PEG 6000 in a molten state, indicating the solubility of CIL in the hydrophilic polymer matrix. However, the drug peak was substantially reduced in intensity in CILSAs (∆H_f_ of 7.6 J/g), indicating a reduction in crystallinity to 16.8% of CIL. The data signify that nearly 83% of the drug was in amorphous form or the molecular state in the hydrophilic polymer matrix [19].

The PXRD pattern of CIL displayed six distinct, well-defined, high-intensity distinct peaks at 2θ values at 11.84° (17044 counts), 12.41° (5945 counts), 14.35° (5452 counts), 16.59° (15133 counts), 18.83° (7916 counts), and 21.86° (9380 counts), indicating the crystalline nature of CIL. The CIL peaks have been reported at 2θ values of 11.80°, 12.17°, 14.40°, 16.43°, 18.85°, and 21.72° in earlier reports, supporting the current findings [6,15,44]. Likewise, the diffractogram of the physical mixture exhibited peaks at 11.82° (8029 counts), 12.41° (3292 counts), 14.38° (3792 counts), 16.58° (7214 counts), 18.83° (5113 counts), and 21.84° (56300 counts). It was observed that the crystallinity of CIL in the physical mixture was decreased to around 47%, indicating the semicrystalline nature of CIL in the mixture. In contrast, the diffractogram of F4 displayed a total of 6 characteristic low-intensity peaks of CIL at 11.82° (1542 counts), 12.41° (1162 counts), 14.38° (1447 counts), 16.58° (1845 counts), 18.83° (1404 counts), and 21.84° (1447 counts). The drug crystallinity was further reduced to about 12% in F4, indicating that 89% of the drug exists in an amorphous or molecular state in the SAs. The DSC and PXRD studies collectively clearly confirm that CIL was embedded in the PEG in a nearly amorphous form. It has been proven by PXRD data that the spherical agglomeration process has the ability to render the solid state of poorly soluble BCS class II drugs more amorphous [45].

The %DE is a surrogate employed to identify how rapidly the drug dissolves in the dissolution medium. The %DE is likely to approach a value of 100% if a pulsatile or burst release is observed. Generally, dissolution efficiency is a factor used to characterize the pulsatile or burst release from dosage forms of therapeutic agents where there would be a need to elicit a faster onset [46,47]. Likewise, the %DE for different batches of SAs was found to range from 37.57 ± 0.08 to 57.01 ± 0.01. Among all the formulations, F4 displayed a significantly better (*p* < 0.001) drug release of 95.50 ± 0.012% at 60 min compared to CIL (37.73 ± 0.89%). The amorphous or molecular state of CIL in the hydrophilic polymer matrix of PEG 6000 is likely to improve the dissolution of the CIL from SAs. In addition, the smaller particle size of the SAs (D90 ~48.85 µm) that would expose a larger surface area to the dissolution media would be another possible reason for better drug release from the SAs. The volume surface mean diameter, found to be 5.78 µm for batch F4, is considered to be pharmaceutically important as it is inversely related to the specific surface. The %DE of CILSAs was found to range from 34.05 ± 0.06 to 57.01 ± 0.01%. The highest %DE was observed with F4, which is composed of PEG-6000, and was found to be significantly higher (*p* < 0.0001) compared to the %DE of CIL (26.27 ± 0.06) as such. PEG 6000 enhances the solubility and dissolution of CIL by acting as a hydrophilic carrier that disrupts crystalline structures through hydrogen bonding, facilitating amorphization and enabling homogenous drug dispersion within the polymer matrix, as demonstrated by the improved performance of F4. Thus, PEG 6000 has the potential to generate a pulsed release of CIL that is likely to saturate the metabolizing enzymes present in the gut wall. The specialized dosage form is likely to overcome the pre-systemic elimination of CIL, thereby enhancing drug absorption and bioavailability. In addition, the SAs composed of PEG 6000 displayed superior compressibility and flow properties. Considering the superior dissolution and %DE, batch F4 of SAs was compressed into ODTs.

The Hausner’s ratio, compressibility index, and angle of repose values indicate good flow characteristics of the blend. The good flow behavior of the SAs can also be attributed to the good flow properties and compressibility of the blend. The lubricated blend could be compressed directly without causing any tablet formulation flaws, such as sticking, picking, lamination, or capping, due to its strong pre-compressional properties. The compressed tablets containing ammonium bicarbonate were rendered porous by subjecting them to vacuum drying. The subliming agent was found to render the tablets porous when subjected to vacuum drying. Ammonium bicarbonate is known to sublime at 36 °C as it decomposes to form ammonia, carbon dioxide, and water [48]. When developing orally disintegrating tablets, ammonium carbonate has been utilized as a subliming agent [49,50].

Bright-field microscopy revealed that the surface topography of ODTs of batches T1 and T3 displayed hollow and well-defined pores, as shown in Figure 8A. In contrast, ODTs of T2 and T4 did not exhibit visible pores, as shown in Figure 8B. The porous ODTs and ODTs of CIL and SAs manufactured by direct compression were found to comply with the permissible pharmacopeial limit (±10%), highlighted in Table 7 [51]. Likewise, the drug content was found to be in the range of 91.06 ± 0.23% to 96.39 ± 0.26% for prepared batches of ODTs [52]. The average weight of ODTs ranged from 66.12 ± 0.02 mg to 73.94 ± 0.08 mg. The uniformity in the weight and drug content was the result of the good flow properties of the lubricated blend. The tablets were compressed enough to impart sufficient hardness to ensure quick disintegration time but, at the same time, adequate mechanical strength. The hardness values for the compressed tablets were found to be 3.2 ± 0.22 kg to 3.5 ± 0.21 kg. The hardness in the range of 3–4 kg in the development of ODTs measuring thickness in the range of 2.48 ± 0.03 mm to 2.70± 0.06 mm would be sufficient to ensure low friability and quick disintegration [53]. The friability values for all batches of compressed ODTs varied between 0.23 ± 0.03% and 0.78 ± 0.08% and were found to be within the permissible pharmacopeial limit and not exceeding 1% [51]. The incorporation of SAs composed of PEG 6000 was found to display good compressibility and produce tablets with good strength. PEG 6000, as a plasticizer, enhances tablet flexibility and reduces brittleness by improving polymer plasticity and reducing intermolecular forces within the tablet matrix.

The directly compressible materials like MCC and mannitol that were incorporated to develop the ODTs are known to improve the compressibility and flowability of the dry blend. In addition, mannitol has a cooling effect and imparts a pleasant mouthfeel when used as a sweetener, while MCC, crospovidone, and croscarmellose sodium serve as super disintegrants. Super disintegrants aid in rapid disintegration without compromising tablet integrity, contributing to overall tablet robustness. In addition, ammonium bicarbonate served as a pore-forming agent in T1 and T3 in the development of porous ODTs [32]. The porous tablets made of CILSAs were found to display significantly short wetting times (*p* < 0.001) compared to the nonporous ODTs. For instance, porous tablets of CIL and SAs exhibited wetting times of 19.82 ± 0.04 s and 6.22 ± 0.03 s, while the nonporous ODTs of SAs and CIL ODTs displayed wetting times of 21.68 ± 0.08 s and 7.61 ± 0.06 s. A comparable wetting time ranging from 11.6 ± 0.57 to 21.42 ± 2.59 s for an ODT was reported in earlier studies [34]. The shorter wetting time recorded can be attributed to the quick diffusion and uptake of water into the microchannels in the porous tablet. The better wettability can be ascribed to the hydrophilic nature of PEG 6000 used for the development of CILSAs. The quick DT observed in the current study was found to depend on the wetting time, as DT was found to decrease with a drop in the wetting time. The porosity of the tablet and the hydrophilic nature of the polymeric carrier are likely to shorten DT. The allowable limit of DT of an ODT determined using a USP disintegration tester must be less than 30 s [54]. DTs of 21.12 ± 0.01 s and 6.26 ± 0.29 s were observed for porous ODTs of CIL and of SAs, respectively, which was significantly shorter (*p* < 0.001) compared to that of nonporous ODTs of CIL and SAs, which exhibited DTs of 25.12 ± 0.06 s and 8.26 ± 0.21 s. The possible explanation may be the incorporation of a subliming agent that led to the formation of microchannels, rendering the tablets porous and promoting water absorption, which, in turn, possibly reduced the DT. The faster disintegration can also be attributed to the incorporation of super disintegrants like MCC, croscarmellose sodium, and crospovidone in the formulation. Super disintegrants are commonly employed to decrease DT by possibly increasing the absorption of water through techniques including swelling and wicking. The super disintegrant croscarmellose sodium has excellent swelling ability and quick water absorption regardless of medium pH. Crospovidone is a hydrophilic cross-linked polyvinyl pyrolidone that is frequently utilized as a super disintegrant. The two super disintegrants have been rationally combined in previous studies to enhance the DT from ODTs [54].

The drug release from the tablets composed of SAs was found to depend on the drug’s solid state and tablet porosity [55]. Similarly, the drug release from porous ODTs of SAs was significantly higher (*p* < 0.0001) compared to the porous ODTs of CIL. The better dissolution profiles of ODTs composed of SAs could be ascribed to the amorphous nature of CIL in the hydrophilic PEG 6000 and the smaller particle size of SAs. Moreover, the porous nature of the ODTs would expedite the diffusion and uptake of the dissolution media into the ODTs, promoting the release of CIL. The channelizing influence of subliming agents in the porous tablets is said to be responsible for the higher release from the porous tablets [56]. Normally, the rate-determining step in ODT dissolution is tablet disintegration. Therefore, the rate at which drugs dissolve is determined by how well ODTs disintegrate. Consequently, the rate at which tablets disintegrate is thought to be a rate-limiting factor that affects the release and absorption of drugs that are poorly soluble [57]. To compare the dissolution profiles of the tablets, the dissolution profile of CILSAs of PEG 6000 was taken as a reference. The dissolution profiles of T3 and CILSAs displayed f1 and f2 values of 12 and 56, respectively, demonstrating a similarity between T3 and CILSAs. Moreover, the drug release from T3 was found to be significantly better (Student’s *t*-test, *p* < 0.005) than the marketed formulation, indicating the superior performance of the SA-embedded porous orodispersible tablets compared to the available marketed formulation. Furthermore, the results imply that the direct compression process does not impact the drug release from T3 when compared to SAs F4. As for patient acceptance, the porous nature of the tablets, coupled with rapid disintegration and enhanced solubility, addresses key patient-centric considerations such as ease of swallowing and improved therapeutic efficacy.

## 5. Conclusions

Agglomerates of Cilnidipine were successfully produced by a spherical crystallization technique using different hydrophilic polymers such as HPMC E 50, PVP K30, and PEG 6000 as carriers. The SEM images revealed the spherical shape and smooth surface. DSC and XRD collectively indicated that the CIL was rendered more amorphous in the hydrophilic carrier. The polymeric SAs-laden ODTs produced by direct compression that were rendered porous by incorporation of ammonium bicarbonate proved to display a short disintegration time and quick release compared to ODTs of CIL. The studies proved that ODTs composed of SAs have the potential to elicit a pulsed dose of CIL at the absorption site in the oral cavity that is likely to evade the pre-systemic gut wall metabolism and increase the drug absorption and, therefore, the bioavailability. Thus, the proposed innovative drug delivery platform with the potential to elicit faster action could mitigate the occurrence of major cardiovascular risks. However, the potential issues related to scalability and large-scale manufacturing need to be addressed during the commercialization of the new platform, considering the number of steps involved. In addition, further in vivo studies are needed to ascertain the enhanced bioavailability and clinical effectiveness of the novel platform developed.

## Figures and Tables

**Table 1:** Composition of formulation of spherical agglomerates.

Ingredients (mg)	F1	F2	F3	F4	F5	F6	F7	F8
Cilnidipine	100	100	100	100	100	100	100	100
GMS	100	-	-	-	50	50	-	50
PVP K-30	-	100	-	-	50	-	50	-
HPMC E50	-	-	100	-	-	50	-	-
PEG 6000	-	-	-	100	-	-	50	50
Talc	20	20	20	20	20	20	20	20

**Table 2:** Master formula for preparation of rapid orodispersible tablets.

Ingredients	T1 *	T2 ^	T3 *	T4 ^
CIL	10	10	-	-
Spherical agglomerates (~10 mg CIL)	-	-	15	15
Directly compressible mannitol	19	24	19	24
Microcrystalline cellulose (Avicel -PH 102)	20	25	20	25
Croscarmellose sodium	4	4	4	4
Cros-povidone	4	4	4	4
Ammonium bicarbonate	15	-	15	-
Magnesium stearate	2	2	2	2
Talc	1	1	1	1
Total tablet weight	75	70	80	80

* Indicate batches T1 and T3 represent porous ODTs; ^ Indicates batches T2 and T4 represent ODTs.

**Table 3:** Fourier transform infrared characteristic peak of CIL, PM, and SAs.

Peak Position (cm^−1^)	Functional Group	Peak Position in CIL (cm^−1^)	Peak Position in PM (cm^−1^)	Peak Position in SAs (cm^−1^)
3000–2800	N-H (Stretch)	3289.0	3288.63	3280.12
1725–1705	C=O (Stretch)	1697.18	1698.16	1689.13
1662–1626	C=C (Stretch)	1648.22	1646.31	1638.34
1550–1475	NO_2_ (Stretch)	1523.49	1522.12	1539.30

**Table 4:** Percentage yield and Drug Content of SA.

Formulations	Percentage Yield	% Drug Content
F1	66.82 ± 0.08	83.65 ± 0.42
F2	82.02 ± 0.06	95.18 ± 0.23
F3	76.12 ± 0.01	94.18 ± 0.12
F4	84.02 ± 0.04	97.49 ±0.29
F5	72.09 ± 0.02	91.26 ± 0.16
F6	73.18 ± 0.06	91.83 ± 0.18
F7	80.08 ± 0.03	94.23 ± 0.02
F8	76.12 ± 0.01	94.03 ± 0.06

**Table 5:** Micromeritic properties of CIL and its SAs.

Formulations	Carr’s Index %	Hausner’s Ratio	Angle of Repose (°)
CIL	33.6 ± 0.08	1.52 ± 0.06	31.82 ± 0.02
F1	14.11 ± 0.01	1.16 ± 0.06	23.60 ± 0.02
F2	13.50 ± 0.06	1.17 ± 0.03	22.40 ± 0.03
F3	14.90 ± 0.08	1.18 ± 0.05	23.20 ± 0.08
F4	11.66 ± 0.01	1.11 ± 0.03	23.01 ± 0.03
F5	13.82 ± 0.02	1.16 ± 0.02	24.20 ± 0.07
F6	14.73 ± 0.03	1.17 ± 0.08	23.08 ± 0.02
F7	14.22 ± 0.02	1.16 ± 0.01	23.30 ± 0.03
F8	12.52 ± 0.03	1.14 ± 0.06	23.60 ± 0.06

**Table 6:** Dissolution efficiency of CIL and its SAs.

Formulations	Dissolution Efficiency (%)
CIL	26.27 ± 0.06
F1	37.57 ± 0.08
F2	56.81 ± 0.03
F3	45.89 ± 0.03
F4	57.01 ± 0.01
F5	36.98 ± 0.05
F6	34.05 ± 0.06
F7	41.37 ± 0.03
F8	40.34 ± 0.01

**Table 7:** Post compressional parameters of prepared orodispersible tablets.

Parameters	ODTs of CIL		ODTs of SAs	
	T1 *	T2 ^	T3 *	T4 ^
Weight variation (mg)	66.12 ± 0.22	68.16 ± 0.26	73.94 ± 0.38	72.35 ± 0.44
Hardness (Kg)	3.2 ± 0.22	3.1 ± 0.23	3.5 ± 0.21	3.4 ± 0.12
Thickness (mm)	2.52 ± 0.07	2.48 ± 0.03	2.70± 0.06	2.58 ± 0.04
% Friability	0.23 ± 0.08	0.26 ± 0.06	0.28 ± 0.03	0.27 ± 0.06
Drug content (%)	93.92 ± 0.18	91.06 ± 0.23	93.12 ±0.13	96.39 ±0.26
Wetting time (sec)	19.82 ± 0.04	21.68 ± 0.08	6.22 ± 0.03	7.61 ± 0.06
Disintegration time (sec)	21.12 ± 0.01	25.12 ± 0.06	6.26 ± 0.29	8.26 ± 0.21
Dissolution (in 60 min.)	37.10 ± 0.37	34.70 ± 0.27	94.16 ± 1.41	84.75 ± 1.69

* Batches T1 and T3 represent Porous ODTs; ^ Batches T2 and T4 represent ODTs.

**Table 8:** Stability data indicating evaluation parameters of the ODTs (T3) in controlled and accelerated storage conditions.

PARAMETERS	0 Months		1 Months		2 Months		3 Months	
	Controlled	Accelerated	Controlled	Accelerated	Controlled	Accelerated	Controlled	Accelerated
Weight variation (mg)	73.96 ± 0.34	73.96 ± 0.34	73.95 ± 0.30	73.93 ± 0.21	73.95 ± 0.18	73.93 ± 0.23	73.91 ± 0.17	73.89 ± 0.21
Hardness (Kg)	3.5 ± 0.23	3.5 ± 0.23	3.5 ± 0.18	3.5 ± 0.13	3.5 ± 0.14	3.5 ± 0.19	3.5 ± 0.12	3.4 ± 0.13
Thickness (mm)	2.70 ± 0.03	2.70 ± 0.03	2.70 ± 0.04	2.70 ± 0.06	2.70 ± 0.03	2.70 ± 0.06	2.70 ± 0.08	2.68 ± 0.08
% Friability	0.28 ± 0.05	0.28 ± 0.05	0.28 ± 0.023	0.28 ± 0.025	0.28 ± 0.72	0.28 ± 0.98	0.28 ± 0.84	0.29 ± 0.95
Drug content (%)	95.12 ± 0.13	95.12 ± 0.13	95.11 ± 0.07	95.07 ± 0.11	94.93 ± 0.11	94.99 ± 0.12	94.90 ± 0.11	93.93 ± 0.13
Wetting time (sec)	6.21 ± 0.04	6.21 ± 0.04	6.22 ± 0.02	6.24 ± 0.01	6.26 ± 0.03	6.29 ± 0.04	6.27 ± 0.02	6.36 ± 0.04
Disintegration time (sec)	6.26 ± 0.24	6.26 ± 0.24	6.27 ± 0.21	6.28 ± 0.11	6.28 ± 0.19	6.29 ± 0.21	6.32 ± 0.14	6.85 ± 0.23
Dissolution (60 min)	94.16 ± 1.34	94.16 ± 1.34	94.14 ± 1.12	94.08 ± 1.30	94.01 ± 1.18	94.03 ± 1.28	93.82 ± 1.12	93.58 ± 1.19

## Data Availability

All data analyzed or generated during this study are included in this article.

## Supplementary Materials

The following supporting information can be downloaded at https://www.mdpi.com/article/10.3390/pharmaceutics17020170/s1, Video S1: wetting of porous ODTs of SAs, Video S2: wetting of ODTs of SAs.
